# Nutrition-Based, High-Protein Adjunct Therapy for Hashimoto's Thyroiditis: A Case Report

**DOI:** 10.7759/cureus.91597

**Published:** 2025-09-04

**Authors:** Savannah L Limbaugh, Lisa A Ennessy, Karen S Davis, Vicki T Allen

**Affiliations:** 1 Clinical Nutrition, Maryland University of Integrative Health, Laurel, USA

**Keywords:** amino acid supplementation, case report, fatigue, high-protein diet, hypothyroidism, leg weakness, hashimoto’s thyroiditis

## Abstract

Hashimoto’s thyroiditis (HT) can cause symptoms as weight gain and fatigue, or with more drastic ones like lowered blood pressure, low resting heart rate, or manifestation of nutritional deficiencies. While diagnostic criteria for HT are aimed at detecting abnormalities in thyroid hormones, there is scarce data on nutritional markers for concerns related to the manifestation of thyroid disease symptoms. We report the case of a 21-year-old overweight female with a 10-year history of HT. The patient developed severe bilateral lower extremity muscle weakness and fatigue, severe enough to restrict exercise activity and normal daily activity, and affect the quality of life. These symptoms were accompanied by relatively normal blood markers for thyroid hormones.

Adjunct therapy consisted of a high-protein diet, utilizing the one-gram per pound of body weight method, supplemented with daily essential amino acids, including tyrosine, valine, and leucine. With nutritional intervention implementation, the patient’s symptoms of weight loss, fatigue, and lack of endurance capacity resolved over two weeks. She was able to restart daily exercise without symptom relapse. The report highlights the resolution of symptoms in an HT patient and delves into the connection between amino acid deficiency and thyroid disease, along with the novel nutritional intervention employed for managing this patient's case.

## Introduction

Hashimoto’s thyroiditis (HT) is an autoimmune condition characterized by the destruction of thyroid tissue, resulting in the underproduction of thyroid hormone and, in some cases, impaired thyroid function, which can lead to hypothyroidism or hyperthyroidism. HT is more commonly diagnosed in women than in men and is typically detected based on elevated thyroid-stimulating hormone (TSH), suboptimal levels of free thyroxine (FT4), and elevated antithyroid peroxidase (TPO) antibodies [1]. While symptoms may be present in patients early in the disease process, labs will indicate normal or within-range parameters. Initial symptoms include, but are not limited to, constipation, fatigue, weight gain, and dry skin; more severe symptoms can include cold intolerance, peripheral neuropathy, low energy, muscle cramps, joint pain, and memory loss. Patients may also present with abnormalities in lab work, such as anemia (30-40%) and abnormal lipid profiles (60-90%) [1]. 

Nutritionally, specific nutrients can be utilized to improve or optimize thyroid function. These include selenium, potassium, copper, magnesium, zinc, iron, and vitamins A, B, C, and D [2]. Additionally, macronutrients, including adequate protein intake, have been associated with improved thyroid function. Protein provides essential and nonessential amino acids, otherwise described as protein building blocks in the body. Tyrosine, an amino acid derived from phenylalanine, can be found in many protein sources and is used to make thyroxine (T4), making it essential for thyroid hormone production. Additional deficiencies in amino acids have been shown to negatively affect thyroid hormone production [3]. We present the case of a 21-year-old female with HT who developed severe muscle weakness and fatigue, which resolved on a high-protein diet and amino acid supplementation. This report adheres to the CARE guidelines [4].

## Case presentation

Presenting concerns

A 21-year-old caucasian female with previously diagnosed HT and hypothyroidism presented to a functional nutritionist due to concerns about fatigue, weight gain, and leg weakness. She had been dealing with symptoms since September 2022 (nine months). The patient was a doctoral student (DCN) and self-employed. She stated that her health concerns were inhibiting activities of daily life and the ability to complete work. The patient was concerned with physical ability, as fatigue and weakness led to the inability to work out or walk for long periods.

Clinical findings

The patient had been diagnosed with HT and hypothyroidism in 2012; she had lived with the condition for 10 years at the time of the current complaint onset. Before developing primary symptoms, the patient had been an avid workout enthusiast. She had been working out five days/week with moderate intensity of strength training, with additional cardiovascular exercise for three days/week, for the past three years. Diet was nutrient-dense with minimally processed foods. She had tracked nutrition intake through a tracking app to ensure adequate nutrition for training. The patient had moved two months before the onset of symptoms and had been unable to exercise during this period due to changes in schedule, availability, and access. During the move, the patient reported less control over diet and often made random meals of what was available or convenient. The patient had also stopped tracking her diet during the move. The patient had reported previous complaints of fatigue associated with HT flare-up or alteration in thyroid hormones, but had never experienced such a degree of fatigue or leg weakness.

Diagnostic focus and assessment

Before starting the intervention, the patient's lab work levels did not indicate that protein levels were inherently deficient in plasma. To further understand her complaints, comprehensive lab work was run over the nine-month intervention period (Table 1). There were no obvious red flags indicated in the lab work that would cause such a severe complaint.

**Table 1:** Comprehensive lab work over nine-month intervention HDL: high-density lipoprotein; LDL: low-density lipoprotein; T3: triiodothyronine; T4: thyroxine; TgAb: thyroglobulin antibodies; TPO: thyroid peroxidase; TSH: thyroid-stimulating hormone

Lab marker	September 14, 2022	February 21, 2023	April 14, 2023	September 5, 2023	Reference Range
TSH, mIU/L	3.47	1.53	0.11	0.81	0.5 – 5
Total T3, ng/dL	93	107	123	52	60 – 180
Free T3, pg/mL	-	3.1	3.7	2.6	2.3 – 4.2
Free T4, ng/dL	-	0.9	1	0.7	0.8 – 1.8
TPO, IU/mL	7 IU/mL	6	-	5	<9
TgAb, IU/mL	-	1	-	0.81	<1
Total cholesterol, mg/dL	229	263	184	-	<200
HDL, mg/dL	65	68	51	-	>50
LDL, mg/dL	148	172	119	-	<100
Triglycerides, mg/dL	65	108	58	-	<150
Glucose, mg/dL	87	91	-	-	60 – 99
Total protein, g/dL	6.8	7.9	-	-	6.1 – 8.1

A nutrition-focused physical exam (NFPE) did not indicate any protein deficiency concerns, nor did it indicate any concerns that would explain the patient's presenting complaints. The patient was suspected of having chronic inflammatory response syndrome (CIRS) by the primary care provider (PCP). The nutritionist did not agree that CIRS could be the sole reason for these concerns, and hence, extensive research was performed to determine the root cause of the problem or possible imbalance leading to symptoms. The nutritionist eventually opted to investigate protein and protein intake as HT patients can experience fatigue and weakness when nutritional protein requirements are not met [3,5].

Diagnosis codes (ICD) for the patient included E06.3 (HT), R53.83 (fatigue), and M62.81 (muscle weakness).

Therapeutic interventions

The intervention implemented for this patient involved incorporating amino acid supplementation, followed by a high-protein diet. After beginning supplementation with amino acids and seeing an almost immediate resolution of symptoms, the nutritionist decided to utilize a high-protein diet with quality protein sources to further recovery and integrate more therapeutic options. Amino acid supplements were dosed at four capsules once a day after breakfast. The supplement included vitamin B6 (14 mg), L-histidine (338 mg), L-leucine (338 mg), alpha-ketoglutarate (300 mg), L-arginine (300 mg), L-lysine (300 mg), L-phenylalanine (300 mg), L-valine (300 mg), L-isoleucine (270 mg), L-methionine (270 mg), and L-threonine (270 mg). 

Protein sources included in the high protein intervention included quality sources of lean ground beef, lean ground bison, chicken, ground turkey, organic Greek yogurt, organic cottage cheese, bone broth, raw milk, isolate protein powder, collagen powder, tuna, and salmon. Protein was dosed at 1 gram per pound of body weight. The patient weighed 138 pounds at the time of intervention, so the aim was 138 to 145 grams of protein daily. To achieve this, protein was provided with a detailed meal plan from a nutritionist, but the patient could also choose to track her protein intake daily to ensure adequate intake.

Follow-up and outcomes

The first visit occurred in February 2023, when the patient presented her concerns. To combat weight gain, fatigue, and leg weakness, the nutritionist worked alongside the patient's PCP to change thyroid medication dosage, and the patient was placed on a 12-week metabolic reset diet. The next visit was in May of 2023; the patient had lost 20 pounds due to a combination of medication change and dietary intervention, but still presented with complaints of leg weakness and fatigue. The PCP decided to run coagulation labs. While waiting for the results of these labs, the nutritionist began researching various intervention options. 

In June 2023, the nutritionist suggested placing the patient on an amino acid supplement once daily after breakfast to determine if it made any changes to symptoms. Within a week, the patient had reported increased energy, lessened fatigue, and leg weakness had decreased. After these initial results, the nutritionist suggested placing the patient on a high-protein diet to help increase protein intake. A meal plan was provided to the patient. The last visit occurred in September 2023; after following the plan for 2.5 months meticulously, the patient reported full resolution of leg weakness and fatigue. Around two weeks into the plan, the patient was able to restart regular exercise and did not suffer any complaints or concerns. The patient will continue on the plan until the next visit, and it will be reassessed at that time. 

At the time of preparing this report, the patient is training for a half-marathon. She can exercise daily with no complaints and does not suffer from leg weakness or fatigue. The patient has reported that she can consume ample amounts of nutrition with no concerns of weight gain. We assessed the compliance of this plan based on self-reported measures, the patient’s nutrition journal, and how the patient reported she was feeling. The patient was dedicated to improving her health and feeling better, so it made it simple to measure adherence. There was a two-to-three-day period when the patient ran out of supplementation and was waiting for delivery. During this time, she did not notice any concerns or complaints of fatigue or weakness. No adverse events were reported.

Patient perspective

“Being young and struggling with unknown fatigue and leg weakness was challenging; I felt like practitioners were pushing my concerns to the side as just normal symptoms of Hashimoto’s. During this period, I was pursuing my Doctorate and starting my own business. I had many moments where I considered withdrawing from my degree and closing my business. Through this experience of utilizing a high-protein diet and amino acid supplementation, I am grateful for the approach that has allowed me to return to normal daily functioning while being able to pursue my goals, like finishing my Doctoral degree and running a half-marathon.”

## Discussion

Patients with hypothyroidism and HT are commonly treated with thyroid hormone replacement from pharmacological sources to replenish or resolve thyroid hormone levels. Despite the resolution in serum thyroid hormone levels, also called euthyroid, many patients report persisting symptoms of thyroid disease. An estimated one-third of individuals presenting with concerns of fatigue have underlying thyroid disease. Fatigue in thyroid patients has been related to alterations in muscle activity, cognitive function, proprioception, and suboptimal exertion [6]. In addition to fatigue, neuromuscular symptoms are common in those diagnosed with altered thyroid function; an estimated 30 to 80% of patients will experience some form of neuromuscular symptoms [7].

A recent study compared euthyroid Hashimoto’s patients (24) to healthy patients without Hashimoto’s (25) to measure the difference in physical fatigue and muscle pain. The study measured physical fatigue with an arm movement test and six-minute walking tests. Each patient was asked to self-report fatigue using the Fatigue Scale for Motor and Cognitive Function. The study found that individuals with Hashimoto’s but with euthyroid levels were, on average, diagnosed with fatigue six years earlier than their healthy counterparts, and the physical fatigue tests found a clinically significant difference between these two population groups (7). In conjunction with neuromuscular symptoms, Hashimoto’s patients will often experience amino acid depletion. Twenty amino acids are known to make up protein in the human body, and all of them play an influential role in transporting and converting thyroid hormones. Most notably, asparagine, glutamine, serine, valine, citrulline, and arginine have been shown to have the most significant and specific effects on thyroid function [8]. 

Deficiencies with asparagine and serine are highly correlated with serum TSH. TSH is a glycoprotein that contains unique asparagine-linked oligosaccharides on the α-subunit. With a deficiency in asparagine, glycoproteins are inhibited and cannot be properly synthesized. Serine, one of the only amino acids to successfully cross the blood-brain barrier, is responsible for modulating the secretion of TSH in the periventricular region. Deficiencies in valine, leucine, and arginine are clinically correlated with serum T4 and FT4, while also being influential in immunological responses. Serine is also associated with FT4, as it increases blood levels of FT4 to transport to target tissues. The most notable amino acids for thyroid function are tyrosine and phenylalanine. A reduction of tyrosine and phenylalanine is directly correlated to altered serum thyroid hormone levels [8]. 

The correlation between thyroid hormone and amino acid deficiencies appears to be bidirectional. A study involving 17 patients with overt hypothyroidism after a thyroidectomy found an increase in serum amino acids after thyroid hormone supplementation. The study concluded that amino acids may be depleted to abnormal thyroid hormone levels, while abnormal thyroid hormone levels may be due to a lack of amino acids. The amino acids with the most significant serum increases were alanine, valine, and Isoleucine [9]. 

While amino acids are often low in Hashimoto’s patients, they are also often deficient in other physiological issues relating to fatigue, such as chronic fatigue syndrome. While some studies have indicated improvements in symptoms after supplementation with amino acids, data regarding supplementation is lacking, especially related to symptom resolution. In comparison, the use of amino acids in athletes to combat fatigue is established in research. In a double-blind placebo-controlled trial of healthy athletes, they were supplemented with amino acids before and during a treadmill trial. The study found a statistically significant increase in time exhaustion with 20 grams of branch-chain amino acids supplemented one hour before exercise [10,11].

Dietary protein is an essential macronutrient needed for Hashimoto’s and hypothyroid patients, as it provides dietary sources of amino acids, as well as acting as a precursor to enzymes and hormones. The hypothalamic-pituitary-thyroid (HPT) axis, responsible for producing thyroid gland regulation and euthyroid function, is highly influenced by nutrition, specifically protein. More specifically, in Hashimoto’s patients, elevated TSH levels are frequent with protein malnutrition. [12,2]. When the diet has low protein intake, the HPT axis activity shows similar patterns to those caused by starvation. This stimulated starvation leads to a decrease in thyroxine-binding globulin, transthyretin, thyrotropin-releasing hormone (TRH), TSH-β gene expression, and circulating thyroid hormone levels. More specifically, in Hashimoto’s patients, elevated TSH levels are frequent with protein malnutrition [3]. 

Research indicates that thyroid patients with low dietary protein intake report lower plasma levels of T3, T4, TSH, TRH, thyroxine, and TTR compared to their healthy counterparts. Low plasma levels of these biomarkers have been directly correlated with higher levels of fatigue and muscle weakness in HT patients [7,13]. In Hashimoto’s patients with elevated or uncontrolled antibodies, the quality of dietary protein becomes essential. Research shows that a high-protein diet with unprocessed protein sources significantly reduces body weight due to abnormal thyroid hormones, as well as improves nutrient status in Hashimoto’s patients. Complete proteins, or those with all essential amino acids, are more beneficial for thyroid patients, as well as contributing to building muscle and improving endurance. Further, complete proteins inhibit iodide oxidation, which commonly happens when thyroid antibodies are present [3]. 

When working with Hashimoto’s or hypothyroid patients, dietary protein intake is a vital consideration to assess. More specifically, amino acid testing can be used to better understand patients’ fatigue complications or to create a tailored dietary nutrition plan. As this case has demonstrated, the use of amino acids and a high-protein diet improved symptoms of extreme fatigue and muscle weakness while also improving thyroid biomarkers. While this intervention was successful in this patient, the authors recommend additional studies with larger cohorts to better understand and analyze the effects of this dietary protocol.

This report has a few limitations, such as relying on self-reported measures of energy, fatigue, leg strength, and exercise tolerance. There was no specific amino acid testing conducted before starting supplementation, which precluded a baseline comparison. Additionally, results of a single case using an individualized approach may not apply to the broader population. 

The strengths of this case report include isolated treatments of supplementation and the demonstration that a high-protein diet improved energy and muscle weakness after pursuing numerous prior interventions of weight loss and adjusting pharmacological measures. This report includes multiple interventions performed in succession, which provides a deep understanding of what can be effective.

## Conclusions

In patients with HT and hypothyroidism, adequate protein and amino acid intake are essential for proper thyroid hormone synthesis. For proper functioning of the thyroid gland, utilizing these nutrients reduces muscle weakness and fatigue, as well as numerous symptoms associated with thyroid dysfunction. Nutrition may be an ideal means to mediate symptoms by focusing on specific pathways that influence thyroid hormone synthesis, such as utilizing tyrosine and phenylalanine to synthesize TSH, T4, and regulate thyroid hormone levels. Although this case report indicated a significant improvement in symptoms, further studies with larger patient populations are warranted to gain deeper insights into this intervention process.
